# Based on Network Pharmacology and Molecular Dynamics Simulations, Baicalein, an Active Ingredient of Yiqi Qingre Ziyin Method, Potentially Protects Patients With Atrophic Rhinitis From Cognitive Impairment

**DOI:** 10.3389/fnagi.2022.880794

**Published:** 2022-06-10

**Authors:** Xueran Kang, Yuxing Sun, Bin Yi, Chenyan Jiang, Xiaojun Yan, Bin Chen, Lixing Lu, Fangze Shi, Yuanbo Luo, Yisheng Chen, Qian Wang, Runjie Shi

**Affiliations:** ^1^Department of Otorhinolaryngology Head and Neck Surgery, Shanghai Ninth People’s Hospital, Shanghai Jiao Tong University School of Medicine, Shanghai, China; ^2^Shanghai Key Laboratory of Translational Medicine on Ear and Nose Diseases, Ear Institute Shanghai Jiao Tong University School of Medicine, Shanghai, China; ^3^Department of Sports Medicine, Huashan Hospital, Fudan University, Shanghai, China; ^4^Department of Central Laboratory, Taian City Central Hospital, Shandong First Medical University and Shandong Academy of Medical Sciences, Taian, China

**Keywords:** atrophic rhinitis (AR), mild cognitive impairment (MCI), common differential proteins of AR and MCI (CDPAM), cytochrome C (CYCS), Yiqi Qingre Ziyin method, molecular dynamics simulation (MDS)

## Abstract

Cognition may be improved by the active ingredients of the Yiqi Qingre Ziyin method in patients with atrophic rhinitis (AR). This study aimed to identify potential targets of the Yiqi Qingre Ziyin method for the treatment of patients with cognitive impairment. Nasal mucosal tissue samples from patients with AR were subjected to proteomic assays, and differentially expressed proteins were obtained. To explore the mechanism of AR leading to mild cognitive impairment (MCI), a differential analysis of AR related differential proteins in the MCI related GSE140831 dataset was performed. Most AR-related differential proteins are also differentially expressed in peripheral blood tissues of MCI, have similar biological functions and are enriched in similar pathways. These co-expressed differential factors in AR and MCI are known as common differential proteins of AR and MCI (CDPAM). Based on the analysis and validation of the random forest, support vector machine and neural network models, CDPAM acted as a diagnostic marker for MCI risk. Cytochrome C (CYCS) was significantly upregulated in the peripheral blood of patients with MCI. The active ingredients in the Yiqi Qingre Ziqin method were obtained and targeted 137 proteins. Among these targeted proteins, *CYCS* belong to the CDPAM set. Molecular docking and molecular dynamics analysis revealed that baicalein, an active ingredient in the Yiqi Qingre Ziyin method, stably targeted the CYCS protein. Results of the enrichment analysis revealed that the up-regulation of *CYCS* expression may have a defensive effect on the cells to resist foreign stimuli. Therefore, baicalein, an active ingredient in the Yiqi Qingre Ziyin method, may prevent the development and progression of MCI by targeting the CYCS protein.

## Introduction

Despite the relatively minor physical damages to the patients, symptoms such as nasal congestion and headache have a greater impact on their daily performance, including work and study, even regular sleep at night, which profoundly disturb some patients. Recent studies show that patients with chronic nasal cavity inflammation had decreased brain connectivity at one of the main functional centers, which plays a central role in cognitive regulation (Jafari et al., 2021). The pathology and pathogenesis of rhinitis sicca are less studied in modern medicine. Rhinitis sicca (RS) and atrophic rhinitis (AR) show similar mucosal ultrastructural changes, whereas the former’s pathological manifestations and symptoms are mild and reversible. Both of them are often considered the same and treated as one disease. Some authors believe that rhinitis sicca, to a certain severity, can develop into AR; thus, these two types of rhinitis are discussed together during this study. Some studies have reported changes in patients’ cognitive levels caused by negative emotions, consequently severely affecting their quality of life (Haug et al., 2009). The more serious the negative emotions are, the more severe the corresponding cognitive impairment gets. Both are positively correlated, showing that breakthroughs in relieving anxiety and depressive symptoms are keys to improving cognitive function in this group of patients (Zufferey et al., 2017), although no systematic studies have revealed the mechanisms of AS and AR affecting the cognitive function of patients.

Cognitive function is an important part of higher cortical functions of the brain, as reflected in various aspects of mental and intellectual activities, such as memory, behavior, attention, speech, abstract thinking, judgment and spatial relations. Numerous clinical trials were observed for the treatment of cognitive function disorders; however, no drugs have been found effective for their treatment (Pei et al., 2020). Mild cognitive impairment (MCI) is an early stage of cognitive decline, where mild cognitive function impairment has occurred but has not yet caused a decline in daily living skills. According to recent epidemiological studies, children with pollen allergies who suffer from rhinitis experience cognitive impairment and have impaired cognitive function (Blaiss and Allergic Rhinitis in Schoolchildren Consensus Group., 2004; Papapostolou et al., 2021). However, it is not well-known that rhinitis is associated with cognitive impairment.

Changes in serum concentrations of specific proteins may serve as predictive markers for impairment or deterioration of cognitive function. Studies showed a cognitive decline in patients with leukoaraiosis is remarkably correlated with S100B/asymmetric DMA (ADMA) levels (Gao et al., 2015), in which S100B (calcium-binding protein B) is responsible for stimulating the expression of pro-inflammatory cytokines, with DMA, asymmetric DMA (ADMA), and arginine/ADMA ratio as remarkable independent predictors of the variability in Mini-Mental State Examination (MMSE) score and Hachinski Ischemic Scale score, two valuable scales for the early screening of Alzheimer’s disease (Fleszar et al., 2019). Therefore, we suggest that the effects of AR and AS on cognitive impairment may be reflected in blood markers that are potential therapeutic targets for preventing cognitive impairment related to rhinitis.

Traditional Chinese medicine (TCM) and its role in the prevention and treatment of cognitive impairment are gaining increasingly widespread attention, which has the advantages of multi-targeting and fewer side effects, thus, is more suitable for long-term use (Cheng et al., 2020; Pei et al., 2020; Li et al., 2022; Liu et al., 2022). For the treatment of cognitive dysfunction, TCM adheres to the principles of “diagnosis and treatment” and “holistic view.” In recent years, certain progress has been made in the exploration of etiology and pathogenesis, disease prevention and drug treatment. Our literature review showed that although many clinical trials have tentatively demonstrated the effectiveness of TCM in improving the clinical symptoms of cognitive dysfunction in elderly people, more reliable and objective evidence-based medical evidence is still needed (May et al., 2009; Wang et al., 2020, 2021). Flavonoids, alkaloids, phenylpropanoids, triterpenoid saponins and polysaccharides, extracted from natural herbs, which often function as antioxidants, anti-inflammatory agents, anti-apoptotic agents and neural regulators, have proven to be effective treatments for dementia. Some of these pharmacological activities are associated with the regulation of nerve growth factor expression, brain-derived neurotrophic factor and glial-cell-derived neurotrophic factor and their related receptors (Oishi et al., 1998; Pang et al., 2016; Jarrell et al., 2018). Ginseng, *Atractylodes macrocephala*, Poria and Licorice, with anti-inflammatory and antioxidant bioactivities, can improve fatigue and reduce nerve function damage (Chen et al., 2016; Shimato et al., 2018; Jin et al., 2019; Yue et al., 2020). Meanwhile, our preliminary study showed that the Yiqi Qingre Ziyin method can effectively relieve AR symptoms, probably through the targeted modulation of neuropeptide-related genes (including DPP4, OPRD1, and OPRM1) (Lu et al., 2022). Therefore, the active ingredient of the Yiqi Qingre Ziyin method may greatly improve the cognitive level of patients with AS and AR.

This study aimed to identify potential targets of the Yiqi Qingre Ziyin method for treating cognitive impairment in patients with AR, providing a strong basis for traditional Chinese medicine for the clinical treatment of AS and AR.

## Materials and Methods

### Participants

This study recruited patients who underwent surgical treatment at the Ninth People’s Hospital in Shanghai, China. Case samples for proteomics in this study were collected from patients clinically diagnosed with AR (three patients) and uncinate process samples from patients clinically treated with nasal polypectomy (three patients) as a control group. Since uncinate process is routinely removed to expose the maxillary sinus intra-operatively, obtaining a sample of uncinate process does not cause additional damage to the patient. All samples included in the case group were confirmed to have AR. Strict information matching is ensured between samples. Specific clinical manifestations are dry nasal cavity, little or no snot, with nasal itching, nasal congestion, nasal burning, nasal bleeding, blood in the snot, throat dryness and other symptoms; dry, congested and loss of normal luster in the nasal mucosa during the examination of the nasal cavity; and mucosal erosion or ulceration, accompanied by atrophy of nasal mucosa and turbinate. All patients were Chinese residents and voluntarily underwent nasal endoscopy by the same surgeon who conducted the study. All patients signed an informed consent form for study participation. The Ethics Committee of Shanghai Ninth People’s Hospital approved this study (approval no. 2017-323-T243) following the Kissinger Declaration.

### Protein Extraction

Nasal uncinate process tissues from patients with AR nasal mucosa or normal nasal polyps were precipitated and added to an appropriate amount of SDT lysis solution, transferred to a Lysing Matrix A tube and homogenized and broken up using an MP homogenizer. Then, the steps of boiling water bath for 10 min, centrifugation at 14,000 *g* for 15 min, supernatant filtration and filtrate collection were completed, respectively. Finally, the BCA method was used for protein quantification. The quality control procedures of the samples were as follows: 20 μg protein was extracted from each sample and added into 6X loading buffer, then bathed in boiling water for 5 min, followed by 12% SDS-PAGE electrophoresis (250 V, 40 min) and Coomassie blue staining.

### Filter Aided Sample Preparation Enzymatic Digestion and Mass Spectrometry

Filter aided sample preparation (FASP) enzymatic digestion was carried out following the steps reported in a previous study (Zhu et al., 2014). Briefly, each sample was obtained from the protein solution, added separately to dithiothreitol, boiled in a water bath and then cooled to room temperature. About 200 μL of UA buffer was added, mixed and centrifuged; 100 μL of IAA buffer (100-mM IAA in UA) was added, shaken and reacted at room temperature; protected from light and centrifuged for 15 min, and the procedure was repeated twice. An NH4HCO3 solution was added and centrifuged, and the procedure was repeated twice. A new collection tube was changed, added with 40 μL Trypsin buffer (4 μg Trypsin in 40 μL and 40 mM NH4HCO3 solution), shaken for 1 min, placed at 37°C and centrifuged, and the filtrate was collected. Peptides were desalted using a C18 Cartridge, lyophilized and re-solubilized by adding 40 μL of 0.1% formic acid solution and were subsequently quantified (OD280). Mass spectrometric analysis was completed by NanoElute chromatography, and samples were separated using a NanoElute system (Bruker, Bremen, Germany) with a nanoliter flow rate. Samples were separated by chromatography and analyzed by mass spectrometry using a timsTOF Pro mass spectrometer (Bruker, Bremen, Germany). The mass spectrometry raw files were processed using Maxquant’s algorithm. The protein sequence database used for this project is Uniprot_HomoSapiens_20387_20210928_9606_swissprot (download link: http://www.uniprot.org). The raw data were saved in text form.

### Subcellular Localization Analysis

Because proteins can only function successfully if they are in the right place, their subcellular localization is important information in investigating protein function. Protein subcellular localization was determined using a prediction software with WoLF PSORT, converting protein sequences into digital localization features and predicting the subcellular localization of proteins using a K-nearest neighbor classifier (Wiśniewski et al., 2009).

### Gene Ontology and Kyoto Encyclopedia of Genes and Genomes Enrichment Analysis

Enrichment analysis of screened proteins was performed using the Kyoto Encyclopedia of Genes and Genomes (KEGG) and Gene Ontology (GO) (Yu et al., 2012; Chen et al., 2021). The top-ranked genes and those with a *p*-value of <0.05 were visualized using R software similarly to previous studies. Enrichment analysis was performed using Fisher’s exact test (FET) to determine whether differentially expressed proteins tended to be significantly enriched in certain functions (*p*-value < 0.05).

### Data Set Acquisition

The MCI dataset is obtained from the GEO database’s GSE140831 dataset and derived from the GPL15988 dataset annotated with HumanHT-12 v4 Expression BeadChip. The GSE140831 dataset contains 1,129 RNA-seq data from the whole blood of 530 healthy participants and 134 patients diagnosed with MCI.

### Composite Compounds and Drug Targets of the Yiqi Qingre Ziyin Method

In this study, chemical compositions of the Yiqi Qingre Ziyin method were determined through the pharmacology platform of the Chinese Medicine Network [including Traditional Chinese Medicine Database, Traditional Chinese Medicine Systems Pharmacology (TCMSP) database and Bioinformatics Analysis Tool for Molecular Mechanism of Traditional Chinese Medicine (BATMAN-TCM)] (Chen et al., 2014; Ru et al., 2014; Liu et al., 2016). The main active ingredients of each drug were extracted under the following screening conditions: oral bioavailability of ≥30% and drug similarity of ≥0.18 (Di et al., 2021). The active ingredient targets of the Yiqi Qingre Ziyin method were obtained from the TCMSP database.

### Molecular Docking and Molecular Dynamics Simulation

Protein structures were obtained from the alphafold2 database (Cramer, 2021). Target proteins were sequentially hydrogenated, charge-added and ligand-root detected using AutoDock. The structures of active ingredients were obtained from the PubChem database and docked with the corresponding proteins using AutoDock 4.2 software after energy minimization (Trott and Olson, 2009; Kim et al., 2021). The above molecular docking pair structures were used as initial structures for molecular dynamics simulations. The ACPYPE server was used to create small-molecular force fields (Sousa da Silva and Vranken, 2012; Kastritis et al., 2014; Kagami et al., 2017). CHARMM was used to describe protein force fields (Van Der Spoel et al., 2005; Nava, 2018). The solvent model in the system is TIP3P, followed by Na+/Cl- used to balance the system charge (dos Santos et al., 2019) NVT and NPT simulations were performed after a slight increase in a system temperature from 0 to 307 K. Finally, molecular dynamics simulations of the complex object system were performed. Finally, a 20-ns MD simulation of the protein-small molecular complex system was performed, with conformations saved every 1 ps and visualization completed with pymol (Seeliger and de Groot, 2010).

### Gene Set Enrichment Analysis

Gene set enrichment analysis enrichment analysis was performed with the clusterProfiler package of R software using “c2.cp.v7.2.symbols.gmt” from the MSigDB Collections dataset, with *Homo sapiens* species (Hung et al., 2012; Yu et al., 2012). Significant differences for GSEA enrichment analysis were defined as adjusted p of <0.05 and false discovery rate (FDR) of <0.25.

### Statistical Analysis

Statistical analyzes and plots were performed using R software (version 3.6.3). Data for prediction models were obtained from the GSE140831 dataset. The prediction model for this study was constructed based on that of previous studies. The random forest (RF) model and support vector machine (SVM) were constructed using R software packages “caret,” “DALEX,” “randomForest,” and “kernlab”. Furthermore, the neural network models were constructed using “neuralnet” and “NeuralNetTools” packages. Their receiver operating characteristic (ROC) curves are drawn, and area under the curve (AUC) values are calculated using the “pROC” package, including “pheatmap” and “ggplot2” used for plotting. Statistical results were expressed as mean ± standard deviation, and two-tailed *t*-tests or analysis of variance was used to analyze differences in two samples, with *p*-values of <0.05 considered statistically different.

## Results

### Distribution and Enrichment Characteristics of Differential Proteins in Atrophic Rhinitis Proteomics

**FIGURE 1:**
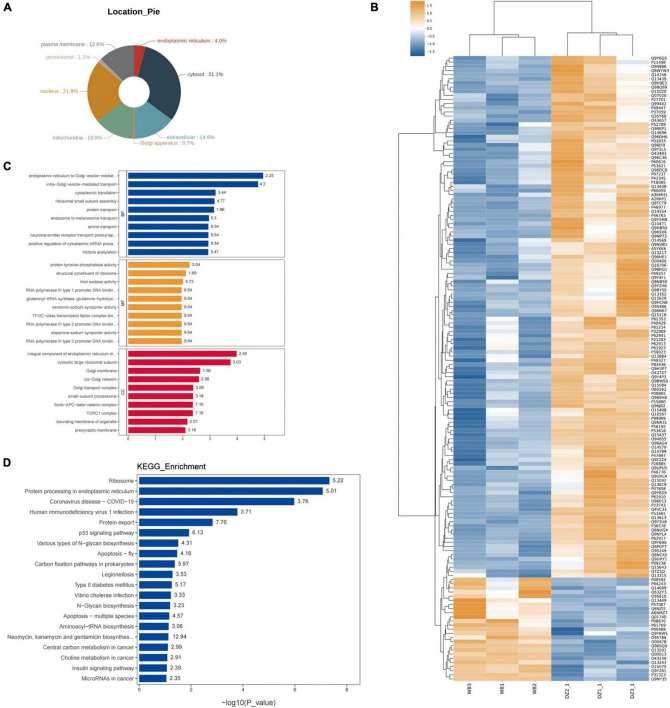
Distribution and enrichment characteristics of differential proteins in atrophic rhinitis (AR) proteomics. **(A)** The pie chart demonstrates tissue distribution of differentially expressed proteins in AR; **(B)** heat map analyzing expression characteristics of all differentially expressed proteins; **(C)** gene Ontology (GO) enrichment analysis of AR-related differentially expressed proteins; and **(D)** Kyoto Encyclopedia of Genes and Genomes (KEGG) enrichment characteristics of AR-related differentially expressed proteins.

In this study, a total of three samples from nasal mucosal tissues of patients with AR and three from unciform processes of patients with nasal polyp were sent for testing. To analyze significant differences in quantitative results, data were screened with at least half of the repeated experimental data within the sample group with non-null values for statistical analysis. Proteins that meet the expression difference ploidy of greater than twofold (up- and down-regulation) and *t*-test of <0.05 screening criteria are considered differentially expressed proteins. In total, 25 genes were obtained with up-regulated expression and 126 genes with down-regulated expression in the nasal mucosa tissue of patients with AR. Subcellular localization analysis showed that their proportion in the subcellular was cytosol (31.1%), nucleus (21.9%), plasma membrane (12.6%), mitochondria (13.9%), endoplasmic reticulum (4.0%), peroxisome (1.3%), and Golgi apparatus (0.7%). Differential genes are enriched in the cytosol, nucleus, plasma membrane and mitochondria (Figure 1A). Heat map analyzes were performed to identify the expression characteristics of all differentially expressed proteins (Figure 1B). Then, the main results of GO and KEGG enrichment were shown in a histogram (Figures 1C,D). During the biological process (BP), functions of enrichment included intra-Golgi vesicle-mediated transport, cytoplasmic translation, ribosomal small subunit assembly, protein transport, endosome to melanosome transport and amine transport. In molecular function (MF), functions of enrichment included protein tyrosine phosphatase activity, structural constituent of the ribosome and thiol oxidase activity. In the cellular component (CC), functions of enrichment included cytosolic large ribosomal subunit, Golgi membrane, *cis*-Golgi network Golgi transport complex, small-subunit processome, Scrib-APC-beta-catenin complex, TORC1 complex, the bounding membrane of the organelle and presynaptic membrane, indicating that AR proteomics differential genes are mainly enriched in protein transfer, DNA transcriptional regulation and Golgi-related functions. Moreover, differential genes have been enriched in the ribosomes, protein processing in the endoplasmic reticulum, coronavirus disease 2019, human immunodeficiency virus 1 infection, protein export and other related pathways, indicating disease progression in AR may be associated with ribosome-mediated protein processing caused by a viral infection, over-activation of export-related functions, and atrophy following functional depletion.

### Atrophic Rhinitis Differential Proteins Are Differentially Co-expressed in the Mild Cognitive Impairment Peripheral Blood

**FIGURE 2:**
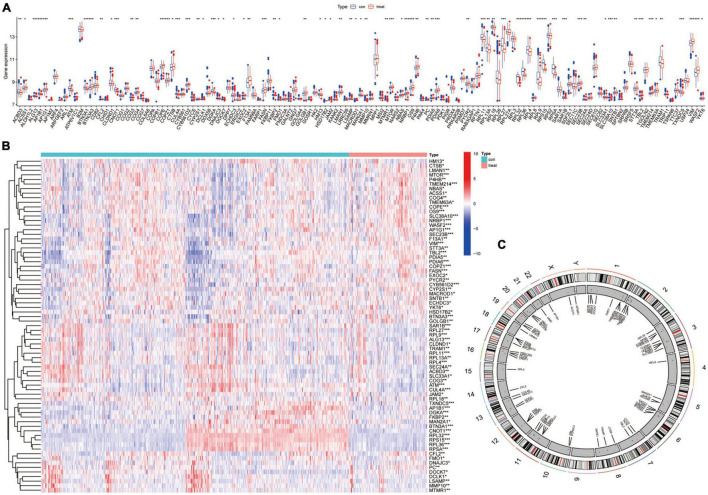
Differential expression and gene distribution in peripheral blood of mild cognitive impairment (MCI) patients. **(A)** An expression box plot shows the AR differential protein-related genes in GSE140831 data; **(B)** heat map demonstrating AR differential protein expressing in the peripheral blood of patients with MCI; and **(C)** circle map demonstrating gene distribution corresponding to AR differential proteins on chromosomes.

**FIGURE 3:**
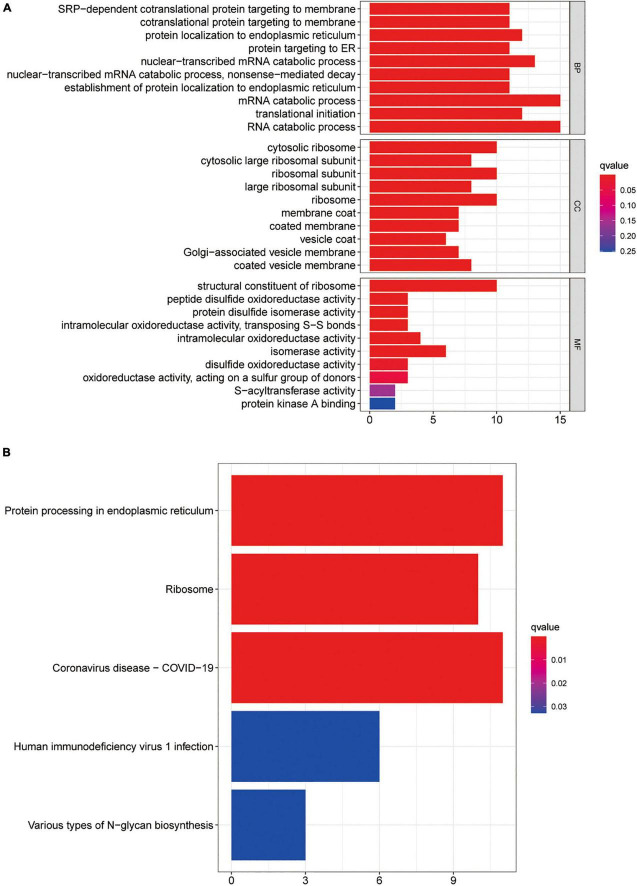
Enrichment analysis of common differential protein of AR and MCI (CDPAM), GO functional enrichment analysis **(A)**, and KEGG pathway enrichment analysis **(B)**.

To explore the mechanism of AR that may lead to MCI, further differential analysis of AR differential proteins in the GSE140831 dataset was performed. As shown in the bar chart, most AR differential proteins were found to be differentially expressed in the GSE140831 dataset (Figure 2A). The top-ranked differential genes were enriched and clustered analyzed in the heat map (Figure 2B). To understand the distribution of these genes on the chromosomes, their expression information was plotted on the chromosomes using the circle diagram (Figure 2C). We referred to these differentially expressed genes (DEGs) co-expressed as common differential proteins of AR and MCI (CDPAM). Enrichment analysis suggests that CDPAM genes are also mainly enriched in the endoplasmic reticulum, ribosome, and vesicle membrane for protein transcription translation and transmembrane transport-related functions and structures; in MF, CDPAM-related genes are mainly enriched in the structural constituent of ribosomes and other related functions, suggesting that disease progression in both AR and MCI is associated with organelle-mediated protein processing, export-related functional hyper-activation, and atrophy after a functional depletion (Figure 3A). In the KEGG enrichment analysis, CDPAM-related genes were enriched during protein processing in the endoplasmic reticulum, ribosome, coronavirus disease 2019, human immunodeficiency virus 1 infection and various types of N-glycan biosynthesis-related pathways (Figure 3B). Here, we found that most AR-related differential proteins were also differentially expressed in the peripheral blood tissues of MCI, and these differential genes have similar biological functions and are enriched in similar pathways, revealing a potential association between AR and MCI.

### Neural Network Model Validating Common Differential Proteins of Atrophic Rhinitis and Mild Cognitive Impairment as Diagnostic Markers for Mild Cognitive Impairment Risk

**FIGURE 4:**
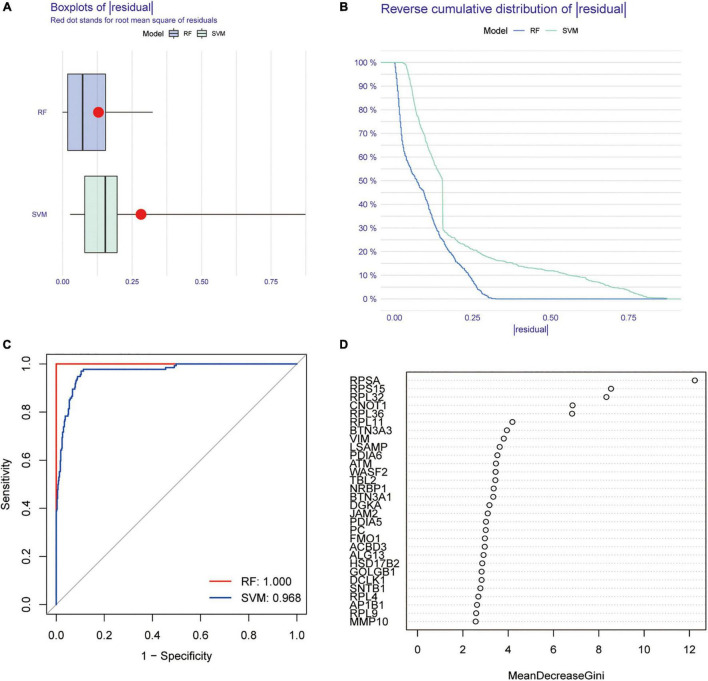
The predictive power of CDPAM. **(A)** Box plots of RF and SVM models in the CDPAM corresponding genes predicting MCI; **(B)** reverse cumulative distribution of RF and SVM models in predicting CDPAM corresponding genes; **(C)** ROC curves of RF and SVM models in CDPAM corresponding genes predicting MCI; and **(D)** weights of each gene in the RF model of CDPAM predicting MCI.

**FIGURE 5:**
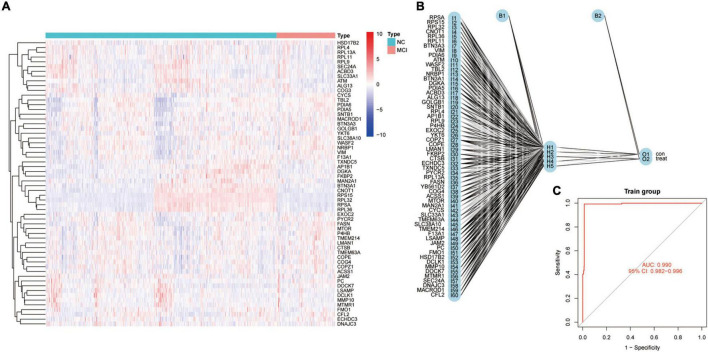
Neural network model validating CDPAM as diagnostic markers for MCI risk. **(A)** Heat map demonstrating gene expression characteristics of building neural network models in the healthy group and patients with MCI; **(B)** model diagram of the CDPAM building neural network models for predicting MCI risk; and **(C)** receiver operating characteristic (ROC) curves of CDPAM corresponding genes predicting the MCI neural network models.

To further evaluate the predictive effects of the CDPAM gene on MCI, MCI prediction models were constructed using RF models and SVM. Box plots and reverse cumulative distribution of RF and SVM CDPAM models corresponding genes predicting the MCI indicate that RF model residual is lesser than that of SVM models (Figures 4A,B). The AUC value of 1 for the RF model of CDPAM corresponding to the gene prediction MCI is slightly higher than that of the SVM model (0.968) (Figure 4C). The relationship between RF model “trees” and “error” is shown in Supplementary Figure 1A. RPSA, RPS15, RPL32, and CNOT1 are among the highest predicted weights in RF models, suggesting that these genes are more closely associated with the MCI risk (Figure 4D). Next, neural network models were used to further validate whether the genes of interest in CDPAM could be used as diagnostic markers for MCI risk with a heat map demonstrating expression characteristics of genes building neural network models in the healthy group and patients with MCI (Figure 5A). Figure 5B shows how to use the model diagram of CDPAM building neural network model for predicting the MCI risk, the AUC value was 0.990 (95% confidence interval: 0.982–0.996) (Figure 5C). Based on the analysis and validation of the RF, SVM and neural network models, CDPAM was found to be a diagnostic marker for MCI risk.

### Molecular Dynamics Simulations Reveal That Baicalein, an Active Ingredient of the Yiqi Qingre Ziyin Method, Can Target and Bind the CYCS Protein

**FIGURE 6:**
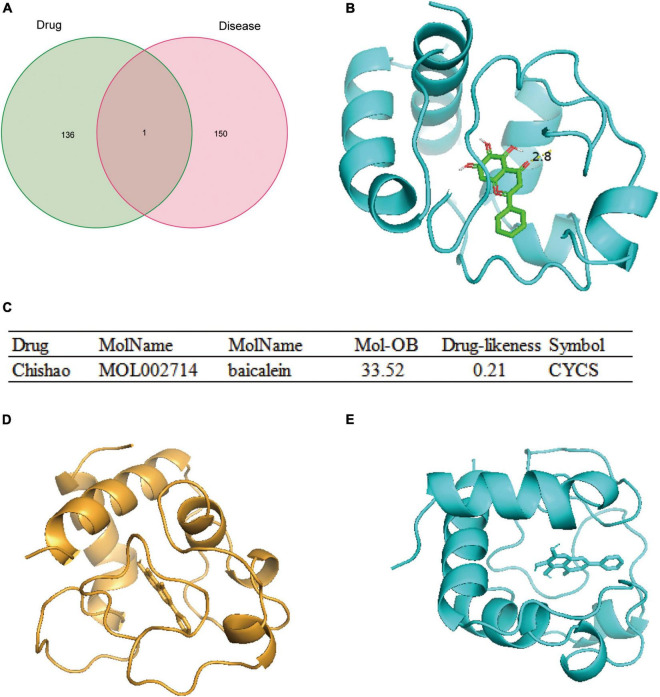
The active ingredient of the Yiqi Qingre Ziyin method – baicalein targeted binding CYCS protein. **(A)** Wayne diagram intersection analysis showing CYCS targeted by the active ingredient of the Yiqi Qingre Ziyin method; **(B)** model diagram of the active ingredient in the Yiqi Qingre Ziyin method – baicalein target-binding *CYCS* protein; **(C)** OB and drug-likeness of the CYCS protein-binding baicalein; **(D)** model diagram of the molecular dynamics simulation in baicalein target binding CYCS protein at 0 ns; and **(E)** model diagram of molecular dynamics simulation in the baicalein target-binding CYCS protein at 20 ns.

Based on traditional Chinese medicine theories, the Yiqi Qingre Ziyin method has been found to control disease progression in AR to some extent in clinical applications. All active ingredients in the Yiqi Qingre Ziyin method were obtained, which targeted 137 proteins, among which the CYCS protein was found to be a protein belonging to the CDPAM set (Figure 6A). A molecular docking analysis was carried out and it was discovered that baicalein, the active ingredient of Yiqi Qinghe method, targets CYCS protein binding (Figures 6B,C). The OB and drug similarity of baicalein, which binds to CYCS protein, can be seen in Figure 6C. Baicalein’s structural diagram is shown in Supplementary Figure 1B. In the Yiqi Qingre Ziyin method, baicalein is the active ingredient of Chishao (Radix Paeoniae Rubra) (Figure 6C). We found that alphafold2 well-predicted the protein structure of CYCS, and the pattern map of the CYCS protein structure is shown (Supplementary Figures 2A,B). Analyzing the binding conformation of baicalein and CYCS protein shows that CYCS forms a hydrogen bond with baicalein at a hydrogen bond distance of 2.8 A (Figure 6B) with a free binding energy of –8.2 (kcal/mol).

**FIGURE 7:**
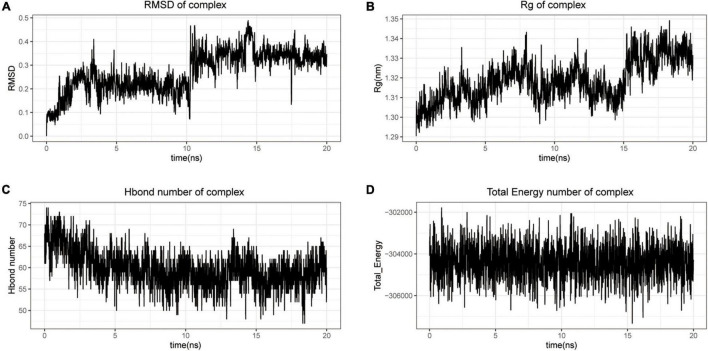
Basic parameters of molecular dynamics simulation. Baicalein-binding CYCS complex RMSD; **(A)** Rg; **(B)** H-bond number; **(C)** total energy; and **(D)** parameter property.

Analysis of ligand–protein interactions before and after MDS revealed that protein conformation contracted to some extent while the binding between CYCS and baicalein was stable (Figures 6D,E). The variation of RMSD values in the CYCS protein-baicalein ligand system with time is shown in Figure 7A. During 0–10 ns, the RMSD values of the system increase due to interactions between the CYCS complex and the solvent; later during 10–20 ns, the RMSD values stabilize due to the interaction between the CYCS protein and the baicalein ligand maintenance. The radius of gyration (Rg) is frequently used to describe variations of the overall structure and can show a relatively stable overall Rg value of the protein (Figure 7B), indicating that the CYCS protein-baicalein ligand system is relatively stable in the structure. Figure 7C shows the relationship between molecular hydrogen bonding and time. Proteins and water molecules form hydrogen bonds, and the figure showed a greater number of hydrogen bonds between water molecules and the protein, with an average of 59.6 hydrogen bonds throughout the simulation. Furthermore, the overall free energy in this system is relatively stable (Figure 7D), suggesting that baicalein stably targets and binds with CYCS protein. By targeting or binding with CYCS protein, baicalein, an active ingredient in Yiqi Qingre Ziyin, may prevent MCI onset and progression.

### Functional Analysis of the Cytochrome C Protein

**FIGURE 8:**
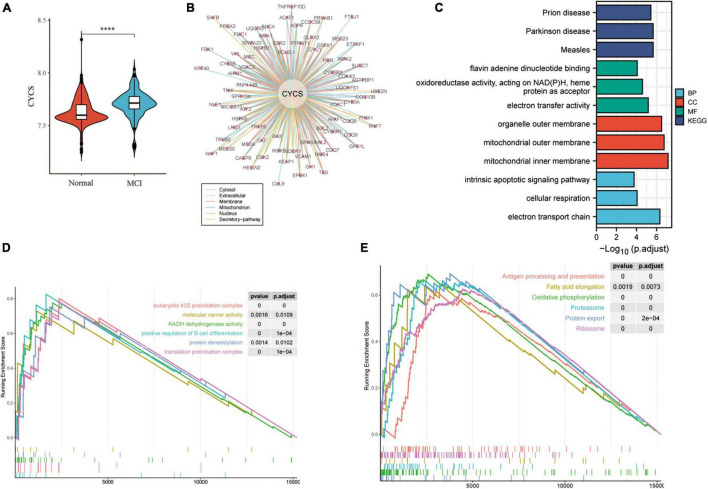
Expression and function analysis of the *CYCS* protein. **(A)** Box plots of *CYCS* differential proteins in the peripheral blood of patients with MCI; **(B)** analysis of *CYCS*-related protein interactions through the OncoBinder model analysis to evaluate the potential function of CYCS; Lower left panel indicates subcellular localization of these protein interactions (*P* < 0.01, Pearson correlation, two-sided); **(C)** GO and KEGG enrichment analysis of proteins interacting with *CYCS*; and **(D,E)** mapping CYCS-related GO functional enrichment in the peripheral blood with GSEA **(D)** and KEGG pathway **(E)** enrichment.

**FIGURE 9:**
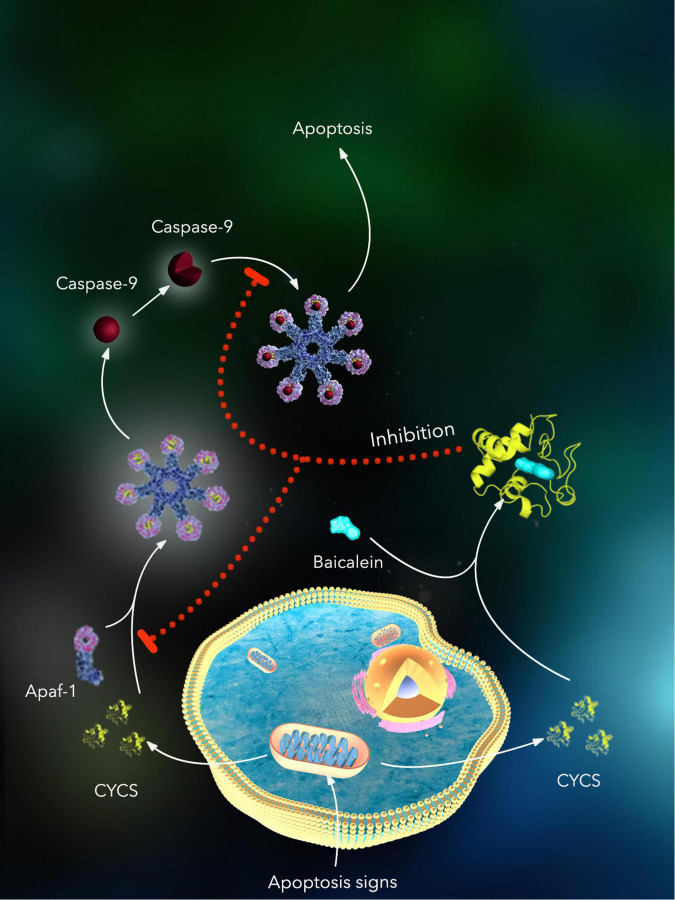
The potential mechanism by which baicalein, the active ingredient in Yiqi Qingre Ziyin method, inhibits the process of apoptosis by targeting the binding of CYCS.

To further evaluate the *CYCS* expression in MCI, the GSE140831 dataset was used to extract CYSC expression data. The *CYCS* expression was significantly upregulated in the peripheral blood of patients with MCI (Figure 8A), indicating a high *CYCS* expression may be associated with the pathogenic process of MCI. The single-cell transcriptome showed that *CYCS* protein was significantly enriched in mouse immune cells (Supplementary Figures 2C,D), suggesting that *CYCS* may be related to the immune cell function. We then analyzed protein interactions associated with *CYCS* through the OncoBinder model to assess the potential function of *CYCS* (Figure 8B), indicating that many proteins interact with *CYCS* in the cytosol, extracellular, membrane, mitochondrion, nucleus and secretory pathway widely enriched in prion disease, Parkinson’s disease, measles, flavin adenine dinucleotide binding, electron transfer activity, organelle outer membrane, mitochondrial outer membrane, mitochondrial inner membrane, intrinsic apoptotic signaling pathway, cellular respiration and electron transport chain (Figure 8C). Moreover, proteins that interact with *CYCS* are associated with the response to and occurrence of viral infections and neurodegenerative diseases. Consequently, these proteins play an important role in membrane function and transmission of the organelle’s oxidative respiratory chain. To assess the relationship between *CYCS* expression changes and cellular pathways and functions, the GSEA was used to map the CYCS-related GO functional enrichment and KEGG pathway enrichment in the peripheral blood (Figures 8D,E). The *CYCS* expression relates to GO function enrichment, such as eukaryotic 43S preinitiation complex, molecular carrier activity, NADH dehydrogenase activity, positive regulation of B-cell differentiation, protein de-neddylation and translation preinitiation complex. GSEA analysis explained the correlation between the *CYCS* and KEGG pathway, including antigen processing and presentation, fatty acid elongation pathway, oxidative phosphorylation, proteasome, protein export, and ribosome pathway. Therefore, *CYCS* expression may be associated with the differentiation and antigen delivery of immune cells, protein export and synthesis and energy metabolism of mitochondria. Based on these results, we suggest that the up-regulation of *CYCS* expression may be a defensive effect made by the cells to resist foreign stimuli, whereas the *CYCS* expression down-regulation may follow the cells that undergo apoptosis. Based on the results of the study, a schematic diagram of the potential mechanism by which baicalein, the active ingredient in Yiqi Qingre Ziyin method, inhibits the process of apoptosis by targeting the binding of CYCS, was drawn (Figure 9).

## Discussion

The cause of AR is complex, and no recognized or effective treatment options in Western medicine currently; therefore, its treatment is mostly local or systemic combination therapy to improve symptoms, which are not satisfactory or prone to recurrence. AR is also likely to results in depression, headache, insomnia, and other symptoms of emotional disorders, causing patients to gradually develop symptoms associated with cognitive impairment. Based on bioinformatics and proteomics analyzes, this study found that most AR-related differential proteins were also differentially expressed in the peripheral blood tissues of MCI, and these differential genes had similar biological functions and were enriched in similar pathways. These findings suggest a potential association between MCI and AR. Furthermore, baicalein, an active ingredient of the Yiqi Qingre Ziyin method, may prevent the development and progression of MCI by targeting the CYCS protein.

The Yiqi Qingre Ziyin method, based on traditional Chinese medicine theory, has been found to control the disease progression of AR to some extent in clinical applications. Baicalein in the Yiqi Qingre Ziyin method may prevent the onset and progression of MCI by targeting and binding with the CYCS protein as our molecular docking analysis revealed. As an active ingredient of Chishao (Radix Paeoniae Rubra), baicalein is derived from the Yiqi Qingre Ziyin method to promote cell proliferation and differentiation, reduce expression of inflammatory factors, protect neuronal cells from death and resist neuronal cell apoptosis. It also reduces apoptotic damage in the brain tissue by upregulating Bax and downregulating Bcl-2 levels (Gu et al., 2016). Modern pharmacological studies have shown that baicalein significantly inhibits NO production and suppresses iNOS and NF-κB protein expressions in LPS-induced BV-2 microglia and primary microglia, thereby suppressing the inflammatory response of glial cells (Suk et al., 2003). Baicalein also blocks NF-κB and MAPK signaling pathways to inhibit the release of inflammatory factors, such as TNF-α, IL-6, and IL-1β, which in turn produces anti-inflammatory activities (Fan et al., 2013). Therefore, the effects of baicalein are suspected to be mediated by targeting CYCS.

Cytochrome C is a protein loosely attached to the surface of the inner mitochondrial membrane and is an important electron transfer chain component. It transfers electrons in the intermembrane space between the adjacent mitochondria. An early event in apoptosis is the release of CYCS from the mitochondria into the cytoplasm. Other proteins involved in the apoptotic process also interact with CYCS. A trend toward an increased percentage of CYCS immunoreactive dystrophic neuropil was observed in pathologically aged control cases compared with AD cases (Woodhouse et al., 2006). Elevated CYCS levels were observed in neuronal bodies and proximal protrusions after multiple *in vivo* experimental injuries (Martin and Liu, 2002; Benjelloun et al., 2003; Page et al., 2003). In a transgenic mouse model of AD, CYCS-positive dystrophic neuron density gradually increased with age until a late disease progression stage when CYCS-positive dystrophic neuron density decreased (Blanchard et al., 2003), suggesting that CYCS upregulation may be a sign that neurons are at an early stage of the apoptotic process. There are two classical apoptotic pathways, the cell surface death receptor pathway (or extracellular pathway) and the mitochondrial-initiated pathway (or intracellular pathway). During apoptosis, the mitochondrial permeability transition pore, MPTP, is overly open and the mitochondrial transmembrane potential is reduced, leading to the release of pro-apoptotic factors such as CYCS from the mitochondria into the cytoplasm. CYCS has been shown to be a key regulator of the mitochondrial apoptotic signaling pathway. Experimental studies have shown that CYCS cannot only directly mediate apoptosis, but also indirectly participate in the apoptotic process by interfering with respiratory chain electron transfer, promoting the production of reactive oxygen radicals, and blocking energy synthesis. CYCS is released from the respiratory chain before being released into the cytosol, and CYCS binds to the WD repeat at the carboxy terminus of Apaf-1, inducing Apaf-1 metamorphosis and further binding to the Caspase-9 precursor, resulting in spontaneous activation of the Caspase-9 complex. The apoptosome continues to activate downstream Caspase-3, triggering a cascade reaction that leads to apoptosis. These results are consistent with those of the current study, in which proteomic results showed reduced CYCS levels in the nasal mucosa of patients with AR, suggesting that their nasal mucosal peripheral nerves are atrophied and in an advanced stage, the reasons for the poor effects of neurotrophic drug therapy. We can speculate that high *CYCS* expression is an early neuronal apoptotic marker and early application of CYCS-targeted drugs for patients with dry rhinitis and AR can effectively inhibit nasal peripheral nerve atrophy and prevent the occurrence of cognitive impairment.

One of the most important elements in the preclinical drug discovery process today is computer-aided drug design (CADD), which facilitates the screening of potential drugs efficiently (Sabe et al., 2021). Out of over 600 relevant studies, there are currently over 70 approved CADD drugs, and this number is steadily increasing (Sabe et al., 2021). To predict the interactions between drugs and proteins, molecular dynamics simulation (MDS) is a reliable method (Honarparvar et al., 2014). Therefore, the binding affinity results obtained by MDS are reliable, and researchers can use this technique to check the validity of molecular docking results (Honarparvar et al., 2014). A number of studies have explored ways to combat cognitive decline using MDS recently. Huperzine A, for example, has been proven to be anti-Alzheimer’s disease, and the study was based on MDS. Moreover, MDS has also been found to have great potential in the treatment of Ebola virus (EBOV) (Darko et al., 2021). As in our study using MDS, DPGS analogs were also able to block interleukin-6 (IL-6) and influence the course of inflammatory diseases (Sanader Maršić et al., 2021). The study identified molecular markers for MCI and identified potential targets for Yiqi Qingre Ziyin formula, which has provided a new example of pharmacological mechanism for MCI and AR treatment in TCM.

This study, based on a neural network model, found that CDPAM can be used as a diagnostic marker for MCI risk. Molecular markers of AR may predict the occurrence of MCI, indicating that MCI and AR may be related. Additionally, molecular dynamics and molecular docking studies indicated that baicalein was capable of stably targeting and binding CYCS protein in CDPAM. CYCS may serve as an intermediate mediator of AR that leads to MCI. However, there are also some limitations to the study. Firstly, a protein sample or blood sample is required for future studies to demonstrate the results of the expression of the target gene or protein. Secondly, considering that the present study is based on a database of clinical proteomic samples, further exploration of the specific mechanisms underlying CYCS preventing MCI occurrence is required. Thirdly, *in vitro* evidence is pending to demonstrate the results of molecular docking and MDS analysis. And there may be a correlation between MCI and AR according to the bioinformatics analysis of this study, though it needs to be investigated clinically. In this process, questionnaire surveys or experimental studies are recommended. Furthermore, the effects of TCM are generally mild and slow (Tomioka, 2017; Pei et al., 2020). Accordingly, if our hypothesis holds true, it is necessary to conduct long-term cohort studies to investigate herbal medicine to prevent the occurrence of AR patients. It is important to note that patients with AS or AR should be guided patiently and informed that their disease is difficult to heal and prone to recurrence, and the efficacy of treatment should not be exaggerated to increase patients’ trust, so as not to cause doctor–patient conflicts due to patients’ eagerness to heal and intolerance to the pain caused by the disease. Simultaneously, a light diet, avoiding spicy and stimulating products, should be considered, and patients should adhere to the medication to obtain better and long-term sustainable results. The current study is limited by objective conditions and further clinical and biological studies are needed in order to further explore the mechanism of MCI prevention after baicalein targeting CYCS. A major contribution of this study was the comprehensive exploration of the mechanism of Yiqi Qingre Ziyin method against MCI, in which the potential link between AR and MCI was also examined. We also explored the potential value of MDS for identifying herbal medicines against MCI and AR.

## Conclusion

Based on differential expression findings of proteomics in AR, this study used neural network models to validate that CDPAM can serve as a diagnostic marker for MCI risk. Molecular dynamics and molecular docking studies finally revealed that baicalein, an active ingredient of the Yiqi Qingre Ziyin method, can stably target and bind with the CYCS protein from CDPAM.

## Data Availability Statement

The datasets presented in this study can be found in online repositories. The names of the repository/repositories and accession number(s) can be found in the article/Supplementary Material.

## Ethics Statement

The studies involving human participants were reviewed and approved by 2017-323-T243. The patients/participants provided their written informed consent to participate in this study.

## Author Contributions

XK conceived and designed the study. RS and YS analyzed the data. All authors were involved to wrote the manuscript and approved the submitted version.

## Conflict of Interest

The authors declare that the research was conducted in the absence of any commercial or financial relationships that could be construed as a potential conflict of interest.

## Publisher’s Note

All claims expressed in this article are solely those of the authors and do not necessarily represent those of their affiliated organizations, or those of the publisher, the editors and the reviewers. Any product that may be evaluated in this article, or claim that may be made by its manufacturer, is not guaranteed or endorsed by the publisher.

## Supplementary Material

Supplementary Figure 1 The relationship between “trees” and “errors” in the random forest model. **(A)** Two-dimensional structure diagram with baicalein **(B)**.

Supplementary Figure 2 Prediction and expression characteristics of CYCS protein in single-cell profiles. **(A)** Structure of the CYCS protein-aligned residue; **(B)** structure forecast of the CYCS protein based on alphafold2; **(C)** annotated single-cell transcript profiles of immune cells in the mouse spleen; **(D)** single-cell annotation map reflecting expression characteristics of CYCS protein in immune cells.
