# Supplementary figures and images for: Based on Network Pharmacology and Molecular Dynamics Simulations, Baicalein, an Active Ingredient of Yiqi Qingre Ziyin Method, Potentially Protects Patients With Atrophic Rhinitis From Cognitive Impairment
Source: Front Aging Neurosci. 2022 Jun 10;14:880794. doi: 10.3389/fnagi.2022.880794 (PMC9226445; doi:10.3389/fnagi.2022.880794)

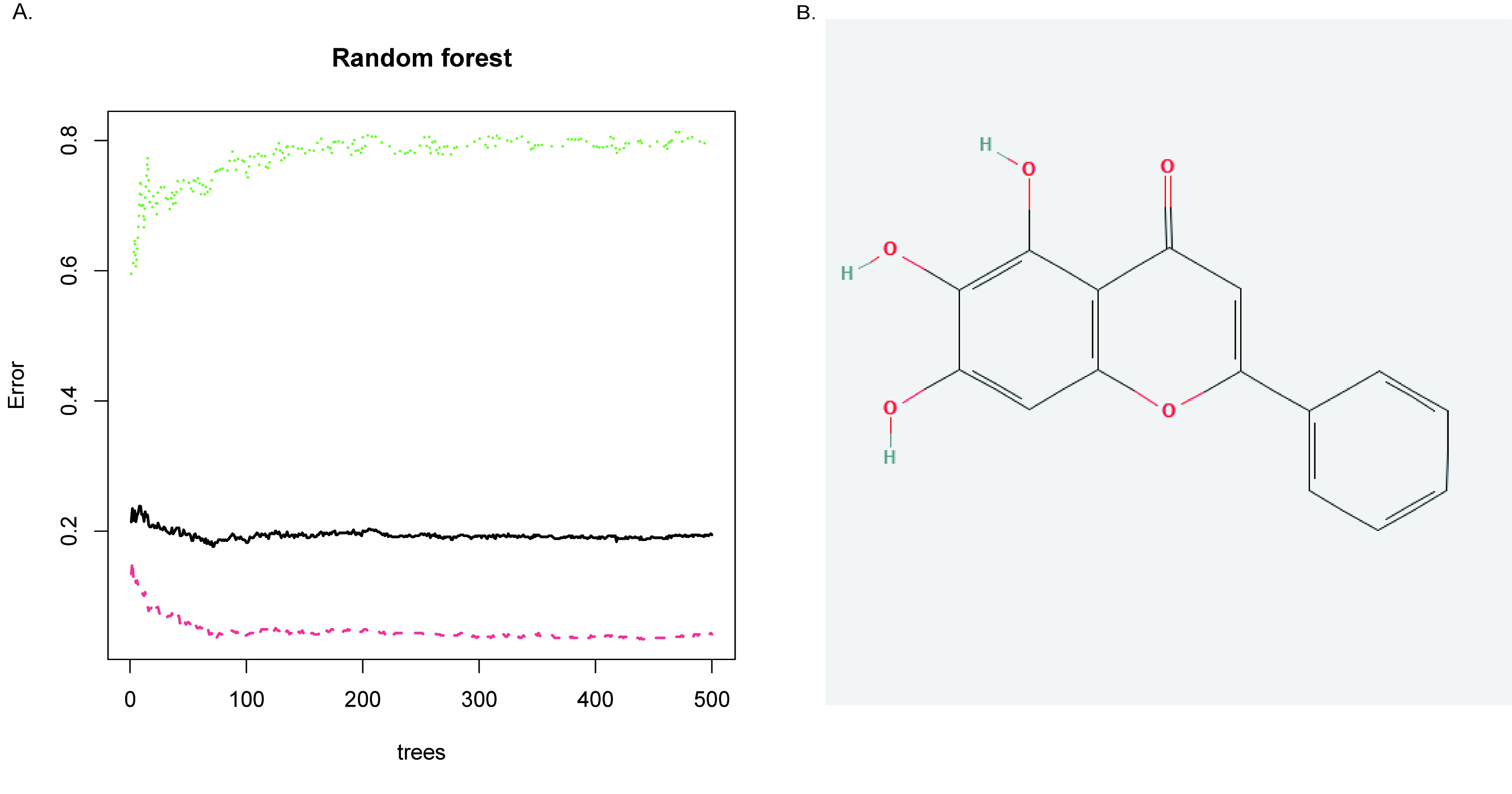

Supplement: Supplementary Figure 1 — The relationship between “trees” and “errors” in the random forest model. (A) Two-dimensional structure diagram with baicalein (B). [file Image_1.TIF]

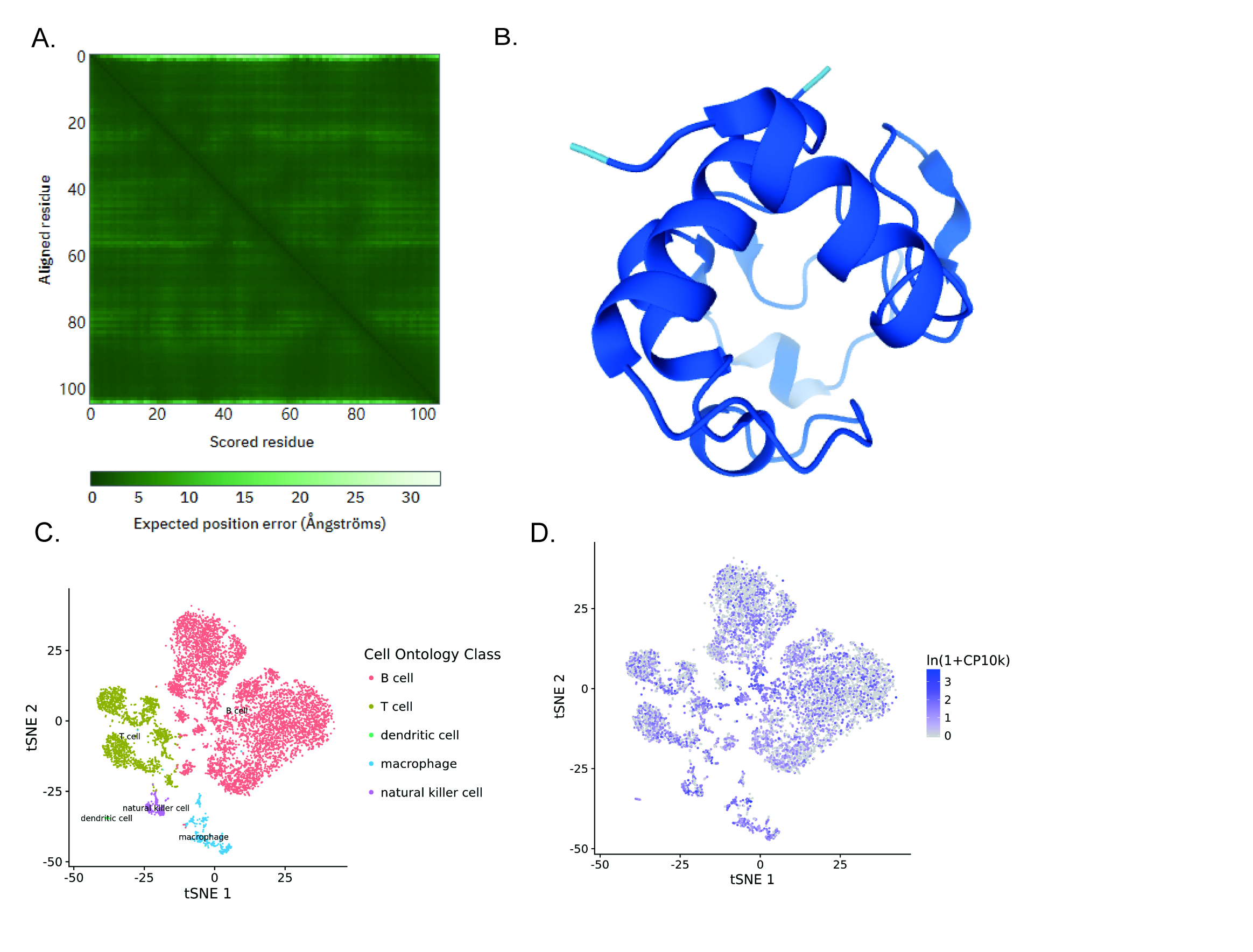

Supplement: Supplementary Figure 2 — Prediction and expression characteristics of CYCS protein in single-cell profiles. (A) Structure of the CYCS protein-aligned residue; (B) structure forecast of the CYCS protein based on alphafold2; (C) annotated single-cell transcript profiles of immune cells in the mouse spleen; (D) single-cell annotation map reflecting expression characteristics of CYCS protein in immune cells. [file Image_2.TIF]
